# Feed intake, emission of enteric methane and estimates, feed efficiency, and ingestive behavior in buffaloes supplemented with palm kernel cake in the Amazon biome

**DOI:** 10.3389/fvets.2022.1053005

**Published:** 2022-12-21

**Authors:** João Maria do Amaral Júnior, Lucieta Guerreiro Martorano, Benjamim de Souza Nahúm, Vinícius Costa Gomes de Castro, Luciano Fernandes Sousa, Thomaz Cyro Guimarães de Carvalho Rodrigues, Jamile Andréa Rodrigues da Silva, Artur Luiz da Costa Silva, José de Brito Lourenço Júnior, Alexandre Berndt, André Guimarães Maciele e Silva

**Affiliations:** ^1^Federal Institute of Amapá, Macapa, Amapá, Brazil; ^2^Embrapa Eastern Amazon, Santarém, Pará, Brazil; ^3^Embrapa Eastern Amazon, Belém, Pará, Brazil; ^4^Federal Rural University of the Amazon, Belém, Pará, Brazil; ^5^Federal University of Tocantins, Araguaína, Tocantins, Brazil; ^6^Postgraduate Program in Animal Science, Federal University of Pará, Belém, Brazil; ^7^Federal University of Pará, Castanhal, Pará, Brazil; ^8^Embrapa Southeast Livestock, São Carlos, São Paulo, Brazil

**Keywords:** by-products, greenhouse gases, methane, nutrition, ruminants

## Abstract

The use of palm kernel cake as an alternative to conventional ingredients, due to the presence of residual fat, can also reduce methane emissions. The objective of the study was to evaluate, in two different experiments, the effects of palm kernel cake supplementation on feed intake, enteric methane production and estimates, and the ingestive behavior of buffaloes in the Amazon biome. In experiment 1, to evaluate feed intake, methane production, and feed efficiency, 20 crossbred females, dry and empty, with a mean age of 34 months and an initial body weight of 514 ± 69 kg, were supplemented with palm kernel cake for 60 days. The supply was calculated in relation to body weight (BW) in four treatments: 0% (control); 0.25, 0.50, and 1% of palm kernel cake, distributed in a completely randomized design. In experiment 2, to evaluate the ingestive behavior, 24 mixed-breed, dry, and non-pregnant buffaloes supplemented with palm kernel cake were evaluated in the less rainy season (LR) and the wettest season (WS) of the eastern Amazon, distributed in a completely randomized in the same treatments as experiment 1. The inclusion of palm kernel cake in the supplementation increased the feed intake of dry matter and components (MM, OM, CP, NDF, ADF, and EE) (*P* < 0.01), reducing the production of enteric methane intake (*P* < 0.01), the ratio per kg of meat produced (*P* < 0.01) and feed efficiency (*P* < 0.01), and influenced the ingestive behavior (time grazing, rumination, and idleness) during the day. We suggest that further research be carried out to verify the results and improve the use of this co-product as a methanogenesis mitigator.

## 1. Introduction

Ruminants, due to enteric fermentation, are known as an important emitter of methane (CH_4_) and contributors to global climate change (1). Methane is one of the main greenhouse gases, and livestock is responsible for about 30% of the anthropogenic production of these gases (2). However, changes in their diet can influence the ruminal microflora, promote better energy use, and reduce methanogenesis and environmental impacts (3).

In this context, agro-industry co-products have been widely studied and present themselves as promising alternatives (4–6). The valorization and use of this material are fundamental for the agri-food sector, as it allows the advancement of the circular economy model with a balance between economic development and environmental and resource protection (7). In addition, they have a low cost compared to conventional ingredients (corn and soy) and an important nutritional composition for ruminant diets.

Palm kernel cake, for example, has ~17% crude protein; 7.7 MJ/kg/MS metabolizable energy; and 60% neutral detergent fiber (8, 9). It is the co-product of palm oil extraction and has between 6 and 16% residual ether extract. In addition to being an important energy source, residual fat promotes the selection of ruminal microorganisms, reducing the population of methanogenic bacteria and energy losses (10, 11).

This is a bioactive function that has been widely studied in animal nutrition because, due to the benefits mentioned, they are possible substitutes for ionophores. The use of these antibiotics in animal feed has been banned in some countries due to the spread of resistance in bacterial communities, which poses some risks to human health (5, 12).

In this context, it is hypothesized that there is a level of palm kernel cake that improves feed intake, does not influence ingestive behavior, but reduces enteric methane emissions. Given the concern about waste disposal, global warming, and the efficiency of diets for ruminants, the objective of this study was to evaluate the effects of palm kernel cake supplementation on methanogenesis, ingestive behavior, and feed efficiency in buffaloes in the Amazon.

## 2. Materials and methods

### 2.1. Ethics committee

The experiment was approved by the Ethics Committee for the Use of Animals (CEUA) of the Universidade Federal Rural da Amazônia (UFRA), under Protocol No 007/2015.

### 2.2. Location

Two experiments were carried out, at the Animal Research Unit “Senador Álvaro Adolfo” (01°25'S e 48°26'W), belonging to Embrapa Eastern Amazon, in the municipality of Belém—PA. According to the Köppen classification, the climate is of type Afi (*A*—tropical humid climates, without a cold season and with the average temperature of the least hot month above 18°C; *F*—Tropical forest climate, with relatively abundant rainfall at all times of the year; *i*—when the amplitude between average temperatures of the hottest month and the coldest month is below 5°C); the average annual rainfall of 3,001 mm is well distributed over the months with the wettest season (WS) from January to June and less rainy (LR) season from July to December. The average annual temperature is 26°C, with relative humidity around 86%.

### 2.3. Experiment 1

For the evaluation of feed intake, emission of enteric methane and estimates, and feed efficiency, were used 20 buffalo females, without a defined racial pattern, dry and non-pregnant, with age and an initial average weight of 32 ± 05 months and 476 ± 39 kg, respectively. The animals, belonging to the herd of Embrapa Eastern Amazon, were distributed in a completely randomized design with 4 treatments (palm kernel cake levels in relation to body weight of 0, 0.25, 0.50, and 1%) and five replications. The values offered were determined from the literature based on the inclusion of co-products in pasture supplements.

Associated with each diet, in all treatments, 0.15% of body weight (BW) of wheat bran was included in order to provide a better acceptability of the supplement. The experiment took place during the months of September and October and used corn silage (CS) as a fodder source.

The animals, confined in individual pens, were adapted for 21 days to experimental diets, facilities, and management, with access to water and mineral mixtures *ad libitum*. The diets were offered in two meals: 50% at 07:00 h and the other 50% at 15:00 h. The amount of roughage offered was weighed daily and adjusted according to the animals feed intake to secure 15% leftovers.

#### 2.3.1. Feed intake and chemical composition

The feed intake of nutritional components was determined by the difference between the amount of each component contained in the feed provided and the amount contained in the leftovers. The chemical composition of the diets was performed at the Animal Nutrition Laboratory of the Federal University of Pará (Table 1). The evaluation of the dry matter (DM), organic (OM), ether extract (EE), and mineral (MM) contents of the ingredients was carried out in accordance with the recommendations of the AOAC (13). The sequential method described by Van Soest et al. (14) was used to determine neutral detergent fiber (NDF) and acid detergent fiber (ADF), and the Kjeldahl method was used to determine crude protein (CP).

**Table 1:** Chemical composition of the ingredients of the experimental diets.

**Fractions**	**Ingredients**		
	**Palm kernel cake**	**Wheat bran**	**Bulky**
Dry matter (%)	90.44	85.85	29.40
Organic matter (g/kg DM)	95.82	93.51	94.92
Crude protein (g/kg DM)	14.27	16.77	7.73
NDF (g/kg DM)	66.30	49.10	56.07
ADF (g/kg DM)	41.49	12.80	31.48
Mineral matter (g/kg DM)	41.8	64.9	50.8
Ether extract (g/kg DM)	12.53	3.64	3.17

DM, dry matter; Bulky, corn silage; NDF, neutral detergent fiber; ADF, acid detergent fiber.

#### 2.3.2. Methane production

Methane (CH_4_) emission was estimated by the sulfur hexafluoride (SF_6_) tracer gas technique (15). A capsule that releases SF_6_ is implanted in the rumen. The gas release rate is known in advance and assumes that the SF_6_ emission standard simulates the CH_4_ emission standard (16). The animals were adapted for 15 days to reduce stress.

A collection and storage diaper in a 60 mm class 20 PVC tube was used, with an internal pressure close to 0 atm, calibrated to reach half an atm pressure at the end of the collection period, using a stainless steel capillary tube with 0.127 mm of inner diameter attached to a halter. The calibration was determined by the length of the capillary tube, which, in this case, was ~4 cm for a period of 24 h.

The diaper was connected to the capillary tube by means of a quick coupling. After the animals had adapted to the sampling apparatus, ruminal gas collections were performed over 5 consecutive days, at 24-h intervals. At 07:00 h 30 min, the animals were removed from the stalls and taken to the management corral, where samples were collected.

The diapers collected were transported to the laboratory, where the samples were diluted and the pressurization was measured until it reached an approximate pressure of 1.2 atm with the injection of nitrogen (the pressure readings were taken on a digital meter ± 0.01). The calibration curves were established using gas standards certified by White Martins/Praxair, with concentrations in ppt (34 ± 9, 91 ± 9, and 978 ± 98 ppt) for SF_6_ and for measuring CH_4_ in ppm (4.85 and 20 ppm), according to Westberg et al. (17).

The CH_4_ and SF_6_ concentrations were determined in a Model 7890A gas chromatograph, equipped with a flame ionization detector (FID), megabore column (0.53 μm, 30 m), Plot HP-Al/M (for CH_4_), electron capture (μ-ECD), and HP-MolSiv megabore column (for SF_6_).

Equivalent emissions were calculated as the ratio between the methane produced and dry matter feed intake, kg of meat produced, feed intake of neutral detergent fiber, feed intake of acid detergent fiber, and feed intake of ether extract.

#### 2.3.3. Feed efficiency

With the total daily dry matter intake (total DMI/kg day) and the average daily gain (ADW), the feed efficiency (FE) of the animals was calculated using the following formula: FE = DWG/total DMI, where total DMI = total daily dry matter intake; DWG = daily weight gain, in kg/day; and FE = feed efficiency.

### 2.4. Experiment 2

#### 2.4.1. Ingestive behavior

The second experiment was carried out, where the evaluations took place in the months of May (wettest season—WS) and September (season less rain—LR). The evaluation took place visually, by the instantaneous scan method, at 5-min intervals for 24 h (13), in 8 periods of 3 h, to determine the time spent in feeding, rumination, and idleness (18).

It was decided to divide the day into 3-h intervals; thus, 8 periods were obtained (11:00–14:00; 14:00–17:00; 17:00–20:00; 20:00–23:00; 23:00–02:00; 02:00–05:00; 05:00–08:00; and 08:00–11:00 h). The data obtained from each 3-h interval was organized as a percentage of the total time. For rumination time, regurgitation, re-chewing and re-swallowing of the feed bolus were accepted. Grazing time included selection, obtaining and handling of feed, and chewing and swallowing the bolus. For the idleness, the time when they were not feeding, eating, or ruminating was counted. For evaluation according to the Amazonian seasons, two observations were carried out, one for LR and another for WS.

### 2.5. Statistical analysis

Feed intake, production of methane and equivalents, and feed efficiency were analyzed by means of the analysis of variance and regression, with the degrees of freedom broken down into linear or quadratic effects, with a significance of up to 5%, using the PROC GLIMMIX of the Statistical Analysis System—SAS version 9.1 (19), according to the statistical model below:


$$\begin{array}{l} Ŷ\text{ij}=\mu +\text{NLi}+\varepsilon \text{ij} \end{array}$$

where *Yij* = value observed in the plot that received treatment *i* in repetition *j*; μ = overall average; *NLi* = fixed effect of oil palm inclusion level *i* (*i* = 0, 0.25, 0.5, and 1%); and ε*ij* = random experimental error associated with each observation assumed NID ~ (0, σ2).

For ingestive behavior: Analysis of variance in a subdivided plot scheme use the model: *Yij* = μ + α*i* + β*j* + αi^*^β*j* + ε*ij*, where *Yij* = observed variables (ingestive behavior); μ = overall mean; α*i* = effect of the *i*-th level of factor α (TP0, TP0.25, TP0.5, and TP1) observed in *Yij*; β*j* = effect of the *j*-th level of the β factor (11:00–14:00; 14:00–17:00; 17:00–20:00; 20:00–23:00; 23:00–02:00; 02:00–05:00; 05:00–08:00; and 08:00–11:00 h); αi^*^β*j* = effect of the interaction α and β; and ε*i* = random error (ε*ij* ~ IN (0, σ2)), with *t*-test at 5% significance level.

For regression analysis: *Y* = α + β*x* + ε, where *Y* = observed variables (feed intake and performance); *x*1 = supply level (TP0, TP0.25, TP0.5, and TP1); α and β = regression coefficients; and ε = random error (ε ~ IN (0, σ2)), using the SAS (19) software.

## 3. Results

### 3.1. Feed intake

The palm kernel cake supplementation showed a linear increase (*P* < 0.01) in the nutrient intake (Table 2). Dry matter intake increased linearly, reaching 7.87 kg/day at the maximum supplementation level (TP1), a value 32.8% higher than the control treatment. This increase in dry matter feed intake reflected in the increase, also linear, of the intake of the other fractions, especially the ADF and EE, which were increased by 56 and 300%, respectively.

**Table 2:** Intake of dry matter and nutrients in female buffaloes supplemented with palm kernel cake.

**Intake**	**Treatments**				**SEM^a^**	***P*** **-value** ^**b**^	
	**TP0**	**TP 0.25**	**TP 0.5**	**TP 1**		***L***	***Q***
DM kg/day^c^	5.92	7.24	7.76	7.87	0.10	< 0.01	0.07
DM (% BW)^d^	1.69	1.97	2.07	2.34	0.02	< 0.01	0.07
DM (kg/BW^0.75^)^e^	54.8	67.1	71.9	72.9	1.02	< 0.01	0.06
MM (kg/day)^f^	0.32	0.36	0.38	0.39	0.01	< 0.01	0.09
OM (kg/day)^g^	5.59	6.87	7.37	7.50	0.27	< 0.01	0.05
CP (kg/day)^h^	0.53	0.67	0.78	0.85	0.03	< 0.01	0.14
NDF (kg/day)^i^	3.26	4.13	4.52	4.65	0.17	< 0.01	0.05
ADF (kg/day)^j^	1.73	2.27	2.54	2.70	0.13	< 0.01	0.06
EE (kg/day)^k^	0.18	0.33	0.44	0.54	0.01	< 0.01	0.14

^a^SEM, standard error of the mean; ^b^L, linear; Q, quadratic; ^c^DM, dry matter; ^d^BW, body weight; ^f^MM, mineral matter; ^g^OM, organic matter; ^h^CP, crude protein; ^i^NDF, neutral detergent fiber; ^j^ADF, acid detergent fiber; ^k^EE, ether extract; TP0, TP0.25, TP0.5, and TP1 are the offers of 0.0, 0.25, 0.50, and 1.0% of palm kernel cake in relation to BW, respectively; PC0.75, metabolic weight. Regression equations: ^c^y = 0.37084 + 0.0511x; ^d^y = 0.0778+ 0.011x; ^e^y = 0.6388 + 0.5015x; ^f^y = 0.024 + 0.0565x; ^g^y = 0.6194 + 0.1285x; ^h^y = 0.105 + 0.01144x; ^i^y = 0.4566 + 0.08137x; ^j^y = 0.3164 + 0.04238; and ^k^y = 0.1162 + 0.0062x.

Crude protein and NDF intakes reached, at the maximum level of cake inclusion, 0.85 and 4.65, against 0.53 and 3.6 kg/day of TP0, respectively. For mineral matter, the increase was 25%, reaching 0.39 kg/day at the maximum level of TP, and organic matter increased from 5.59 (TP0) to 7.49 (TP1).

### 3.2. Enteric methane production and feed efficiency

Enteric methane production decreased linearly with increased TP inclusion in the supplement (*P* < 0.01). In the control treatment, the production of CH_4_ kg/year was 66.04, compared with 26.43 in the supply of 1% of palm kernel cake (Table 3).

**Table 3:** Enteric methane production, feed efficiency, and estimates of emissions in female buffaloes supplemented with palm kernel cake.

**Items**	**Treatments**				**SEM^a^**	***P*** **-value** ^**b**^	
	**TP0**	**TP0.25**	**TP0.5**	**TP1**		***L***	***Q***
Enteric methane^c^	66.04	52.17	54.44	26.43	5.54	< 0.01	0.22
Feed efficiency^d^	0.43	0.39	0.35	0.32	0.03	< 0.01	0.96
CH_4_/meet^e^	0.67	0.55	0.48	0.29	0.01	< 0.01	0.59
CH_4_/DMC^f^	0.03	0.02	0.02	0.01	0.01	< 0.01	0.48
CH_4_/NDFC^g^	0.06	0.04	0.03	0.02	0.01	< 0.01	0.75
CH_4_/ADFC^h^	0.11	0.07	0.06	0.03	0.01	< 0.01	0.61
CH_4_/EEC^i^	0.97	0.43	0.34	0.13	0.07	< 0.01	0.06

^a^SEM, standard error of the mean; ^b^L, linear; Q, quadratic; TP0, TP0.25, TP0.5, and TP1 are the offers of 0.0, 0.25, 0.50, and 1.0% of palm kernel cake in relation to BW, respectively. Regression equations: ^c^y = −11.656 + 2.684x; ^d^y = −0.0375 + 0.009x; ^e^y = 0.0121 + 0.0027x; ^f^y = −0.00478 + 0.000856x; ^g^y = 0.0123 + 0.00184; ^h^y = −0.024 + 0.0035; and ^i^y = −0.0375 + 0.037.

Feed efficiency and estimates of emissions (methane production/kg of meat produced, production methane/DM feed intake, production methane/DNF feed intake, production methane/ADF feed intake, and production methane/EE feed intake) were also lower when the inclusion level of PT was higher (*P* < 0.05). As highlighted, the methane emission per kg of meat produced is 131% lower when the inclusion of TP in the supplementation is 1%.

### 3.3. Ingestive behavior

There was no interaction between the levels of palm kernel cake (*P* > 0.05); however, the periods of day influenced the rumination and idle time (*P* < 0.05). The longest feeding times occurred in the periods between 14:00–17:00, 23:00, and 02:00 h, but there was no difference between them (Table 4).

**Table 4:** Grazing, idleness, and ruminating time in female buffaloes supplemented with palm kernel cake during the less rainy season (LR).

**Periods**	**Treatments**				**Averages**
	**TP0**	**TP0.25**	**TP0.5**	**TP1**	
**Grazing time (coefficient of variation** **=** **37.40%)**					
11–14 h	20.0 ± 6	23.3 ± 12	25.0 ± 12	15.0 ± 8	20.8de ± 10
14–17 h	113.3 ± 32	113.3 ± 12	113.3 ± 16	95.0 ± 22	108.7a ± 27
17–20 h	51.6 ± 12	51.6 ± 18	70.8 ± 17	23.3 ± 12	49.3bc ± 23
20–23 h	29.1 ± 10	28.3 ± 26	32.5 ± 16	33.3 ± 14	30.8d ± 25
23–02 h	122.5 ± 27	112.5 ± 13	92.5 ± 23	114.1 ± 16	110.4a ± 17
02–05 h	6.6 ± 7	3.3 ± 5	10.0 ± 8	9.1 ± 7	7.2e ± 21
05–08 h	65.0 ± 19	56.7 ± 27	62.5 ± 32	65.8 ± 28	62.5b ± 35
08–11 h	31.6 ± 25	35.0 ± 38	50.0 ± 30	30.0 ± 7	36.6cd ± 31
Means	55.0 ± 44	53.0 ± 43	57.0 ± 38	48.2 ± 38	–
**Idle time (coefficient of variation** **=** **40.12%)**					
11–14 h	107.5aA ± 14	129.1aA ± 17	104.1aA ± 14	130.3aA ± 14	117.9a ± 19
14–17 h	15.8bA ± 14	29.1bA ± 19	32.5bA ± 32	59.1bA ± 21	34.1de ± 27
17–20 h	17.5bA ± 11	20.0cA ± 10	15.0bA ± 11	58.3bA ± 23	27.7e ± 23
20–23 h	70abA ± 14	43.3bA ± 15	55.8abA ± 21	82.5abA ± 31	62.9c ± 25
23–02 h	24.1bA ± 18	33.3bA ± 16	20.8bA ± 20	20.8bA ± 15	24.7e ± 17
02–05 h	65abA ± 15	48.3bA ± 25	53.3abA ± 27	42.5bA ± 8	52.2dc ± 21
05–08 h	52.5bA ± 36	73.3bA ± 41	60.0abA ± 35	38.3bA ± 24	56.0c ± 35
08–11 h	96.6aA ± 25	105abA ± 32	88.3aA ± 46	97.5abA ± 21	96.8b ± 31
Means	56.1 ± 38	60.2 ± 43	53.7 ± 39	66.2 ± 39	–
**Rumination time (coefficient of variation** **=** **31.23%)**					
11–14 h	52.5bA ± 17	27.5bA ± 15	50.8bA ± 19	34.1bcA ± 14	41.2d ± 19
14–17 h	50.8bA ± 21	37.5bA ± 18	34.1bA ± 29	25.8cA ± 23	37.0d ± 23
17–20 h	111aA ± 13	108aA ± 20	94.1abA ± 15	98.3abA ± 23	103ab ± 18
20–23 h	80.8abA ± 6	108aA ± 22	91.6abA ± 18	64.1bcA ± 21	86.2b ± 23
23–02 h	33.3bA ± 24	34.1bA ± 15	66.6bA ± 20	45bcA ± 19	44.7cd ± 23
02–05 h	108abA ± 17	128aA ± 27	116.6aA ± 28	128.3aA ± 9	120.4a ± 22
05–08 h	62.5bA ± 22	50.0bA ± 21	57.5bA ± 20	75.8bA ± 23	61.4c ± 22
08–11 h	51.6bA ± 29	40.0bA ± 23	41.6bA ± 32	52.5bcA ± 20	46.4cd ± 2
Averages	68.8 ± 32	66.7 ± 43	69.1 ± 35	65.5 ± 37	–

TP0, control treatment; TP0.25, palm kernel cake 0.25%; TP0.5, palm kernel cake 0.50%; TP1, palm kernel cake 1.00%. Averages in the same column and the same row, within each variable, followed by different uppercase and lowercase letters, respectively, differ from each other (P < 0.05) by Tukey's test.

The time spent for idleness was influenced by the periods of the day, being higher overnight, with an average of 56.04, 96.88, and 117.91 for the periods 05:00–08:00, 08:00–11:00, and 11:00–14:00 h, respectively (*P* < 0.05). Rumination time was also influenced only in the periods of the day (*P* < 0.05), with a shorter time observed in TP1 animals, from 11:00 to 17:00 h.

The highest means of rumination occurred from 02:00 to 05:00 h, with no differences between treatments and an average of 120.42, reducing at times of higher feeding frequency. The times of lowest rumination occurred between 11:00 and 17:00 h, time of intense feeding or idleness.

At dawn and early morning, there was an effect for the grazing time variable (*P* < 0.05). The longest feeding times occurred between 14:00–17:00 (rainy time), 20:00–23:00, and 2:00–5:00 h (milder temperature) (Table 5). During the WS period, there was an effect for idleness between 11:00 and 14:00 h, reaching an average of 126.66 (*P* < 0.05).

**Table 5:** Grazing, idleness, and ruminating time in female buffaloes supplemented with palm kernel cake during the less rainy season (MAC).

**Periods**	**Treatments**				**Averages**
	**TP0**	**TP0.25**	**TP0.5**	**TP1**	
**Grazing time (coefficient of variation** **=** **37.41%)**					
11–14 h	2.5fA ± 4.1	8.3gA ± 10.6	10.8dA ± 10.6	8.3cdA ± 10.3	7.5de ± 8.9
14–17 h	77.5bA ± 10.3	74.1bA ± 12	94.1aA ± 18.8	81aA ± 4.9	81.7a ± 14.0
17–20 h	40 ± 4.4dA	47.5 ± 6.8dA	47.5 ± 8.8cA	43 ± 6.7bA	44.5c ± 7.2
20–23 h	95aA ± 9.4	84.1aA ± 13.9	103.3aA ± 19.1	82aA ± 11.6	91.1a ± 15.7
23–02 h	15.8eA ± 13.5	22.5fA ± 4.1	11.6dA ± 5.5	14cA ± 3.7	16d ± 8.73
02–05 h	57.5cB ± 16.9	60.8cAB ± 8	73.3bAB ± 12.1	80aA ± 10.4	67.9b ± 14.8
05–08 h	69.1bA ± 16.8	37.5eB ± 19.1	49.1cAB ± 11.5	35bB ± 10	47.7c ± 19.5
08–11 h	0fA ± 0	1.6gA ± 4	8.3dA ± 4	0dA ± 0	2.5e ± 4.4
Means	42.9a ± 33.4	49.8b ± 37.7	42a ± 30.1	44.6a ± 35.4	–
**Idle time (coefficient of variation** **=** **18.37%)**					
11–14 h	115.8 ± 34.1	116.6 ± 42. 9	126.6 ± 21.8	147.5 ± 38.4	126.6a ± 35
14–17 h	84 ± 15.9	55.0 ± 24.9	65 ± 21.6	74.1 ± 27.8	69.5b ± 24
17–20 h	16 ± 8.0	11.6 ± 11.2	19.1 ± 15.9	23.3 ± 19.4	17.5e ± 14
20–23 h	65 ± 16.4	37.5 ± 23.8	50 ± 28.1	44.1 ± 13.5	49.1cd ± 22
23–02 h	43 ± 16.6	59.1 ± 20.3	35.8 ± 15.9	44.1 ± 20.8	45.5d ± 19
02–05 h	47 ± 16.0	50.8 ± 22.4	51.6 ± 23.1	38.3 ± 23.5	46.9d ± 20
05–08 h	75 ± 20.2	47.5 ± 17.5	78.3 ± 28.9	60.8 ± 20.3	65.4bc ± 24
08–11 h	40 ± 30.9	35 ± 27.3	45 ± 23.4	17.5 ± 14.7	34.3d ± 25d
Means	60.7 ± 35.3	51.6 ± 36.9	58.9 ± 37.3	56.2 ± 44.5	–
**Rumination time (Coefficient of variation** **=** **36.74%)**					
11–14 h	55.8 ± 28.8	52.5 ± 42.2	45 ± 24	30.0 ± 39.5	45.8 ± 34c
14–17 h	15 ± 13.4	30.8 ± 15.6	40.8 ± 20.8	28.3 ± 33.5	28.7 ± 23cd
17–20 h	121 ± 11.1	120.8 ± 5.8	113.3 ± 15.3	116.6 ± 18.8	117.9 ± 13a
20–23 h	33 ± 22.05	39.1 ± 28	45.8 ± 25.1	40.8 ± 19.3	39.7 ± 23cd
23–02 h	123 ± 15.6	109.2 ± 24.7	121.7 ± 17.2	120 ± 25.3	118.4 ± 20a
02–05 h	53 ± 19.13	55.8 ± 24.9	67.5 ± 18.6	84.1 ± 15.3	65.1 ± 22b
05–08 h	70 ± 19.75	83.3 ± 13.6	64.1 ± 25.9	50 ± 23.6	66.8 ± 23b
08–11 h	20 ± 30.98	16.6 ± 25.8	13.3 ± 21.6	42.5 ± 14.7	23.1 ± 25d
Averages	61.3 ± 44.0	63.5 ± 42.1	63.9 ± 40.1	64 ± 42.5	–

TP0, treatment control; TP0.2, palm kernel cake 0.25%; TP0.5, palm kernel cake 0.50%; TP1, palm kernel cake 1.00%. Averages in the same column and the same row, within each variable, followed by different uppercase and lowercase letters, respectively, differ from each other (P < 0.05) by Tukey's test.

Except between 23:00 and 02:00 h, there was also an effect of the periods on the rumination time (*P* < 0.05), being the period 17:00–20:00 h different from the others. The lowest rumination averages occurred from 8:00 to 11:00 h and from 14:00 to 17:00 h, but there was no difference between them.

## 4. Discussion

### 4.1. Feed intake

It is recommended not to exceed 7% lipid in ruminant diets, as despite the benefits, it can reduce fiber digestibility and feed intake (20, 21). Supplementation with up to 1% of palm kernel cake increased the lipid content of the diets and feed intake. This was possibly due to an increase in ADF and lignin levels and, consequently, reduced digestibility (6, 22). Thus, to meet the nutritional requirements, animals are compensated with increases in DMI and dietary nutrients (23). However, it is worth mentioning that the increase in ether extract, ADF, and lignin in the supplement was not enough to limit feed intake.

### 4.2. Methane production

Alternative ingredients such as co-products, secondary plant compounds, essential oils, and lipids still show inconsistent results in mitigating methanogenesis, ranging from substantial reduction (24–26), to stability or a modest increase in enteric emissions (27–29).

These results are probably due to the composition of the diet and, mainly, to the differences in the lipid source of the ingredient (28). There are fatty acid profiles that are more effective in reducing CH_4_, making studies with alternative feeds even more important (2, 30).

Thus, the inclusion of palm oil cake, whose main fatty acids are oleic, myristic and palmitic (9), and which have an inhibitory action on GHG production (31, 32), endorse the results obtained.

At levels above 0.50% (PC), the inclusion of palm kernel cake in the diet of female buffaloes had a bioactive function and reduced the enteric methane production of the animals, with values below the average emission in buffaloes estimated at 55.00 kg/year (33).

This result, when compared to the effectiveness of ionophores, once again shows promise, since the reduction between the most distant treatments was 40.02%, higher than most studies with antibiotics (34). Ionophores are intended for the control of proteolytic rumen bacteria; thus, they present more modest reductions in terms of methane mitigation, appearing in the results only as a co-benefit of their use (34, 35). Furthermore, the mitigation of CH_4_ by these antibiotics, unlike lipid sources, is reduced in the short term by the selection of more resistant microorganisms, posing a threat to public health (36, 37).

### 4.3. Feed efficiency and estimates of productions

The reduction in feed efficiency was probably due to the increase in ADF and lignin in diets with PT, allied to the increase in DMC. Acid detergent fiber intake among the most distant treatments was 1.56 times higher, reducing digestibility and causing a compensatory increase in feed intake. Furthermore, the increase in EE intake can lead to a reduction in NDF digestibility, reflecting a 34% drop in feed efficiency.

However, the increase in EE intake with the inclusion of PT had a bioactive effect on methanogenic rumen microorganisms, reducing not only the production of enteric methane but also the amount emitted per kilogram of meat produced. This is an important indicator of the efficiency of PT inclusion for the production of more environmentally friendly meat (38, 39), and it has been widely studied (40–42).

The inclusion of the cake in the supplementation, through modulations in the rumen environment, also allowed lower CH_4_ emission per kilogram of DM, NDF, ADF, and EE consumed, demonstrating its efficiency as a bioactive compound in ruminant diets in the mitigation of greenhouse gases (GHG).

### 4.4. Ingestive behavior

The time spent on rumination, feeding, and idleness is directly influenced by diet (43). The nature of the diet, especially the fiber quality, is a determining factor in the oscillation of rumination time and, consequently, feeding, and idleness (44).

Supplementation with concentrates, in turn, as well as weather conditions, also influence the times chosen to perform each activity. Thus, as the availability and quality of the pasture and the inclusion of supplements were common to all, the ingestive behavior of the animals was not influenced. However, the evaluated climatic conditions (LR and WS) influenced the distribution of activities in the periods of the day.

Possibly, this is due to the supply of supplementation and the strategies adopted by the animals to overcome situations of intense heat or rain (45). Immediately after the offer, the animals start feed intake, and, in reflex, the rumination activity is also influenced since it occurs soon after ingestion.

## 5. Conclusion

Supplementation with palm kernel cake is a promising alternative. Its inclusion in the diet increases feed intake, reduces enteric methane production, and improves the proportion of methane per kg of meat produced. We suggest that further research be carried out to verify the results and improve the use of this co-product as a methanogenesis mitigator.

## Data availability statement

The raw data supporting the conclusions of this article will be made available by the authors, without undue reservation.

## Ethics statement

The animal study was reviewed and approved by the Ethics Committee for the Use of Animals (CEUA) of the Universidade Federal Rural da Amazônia (UFRA), under Protocol N° 007/2015. Written informed consent was obtained from the owners for the participation of their animals in this study.

## Author contributions

Experiment design: ALS, AB, JL, and AS. Experiment performed: VC, JA, and LS. Data curation: LM, BN, TR, AS, and JL. Formal analysis: ALS, JS, AS, and AB. Writing-original draft: AS, JL, TR, and JS. All authors contributed to the article and approved the submitted version.
